# Psychosocial factors associated with postpartum psychological distress during the Covid-19 pandemic: a cross-sectional study

**DOI:** 10.1186/s12884-020-03399-5

**Published:** 2020-11-18

**Authors:** Luca Ostacoli, Stefano Cosma, Federica Bevilacqua, Paola Berchialla, Marialuisa Bovetti, Andrea Roberto Carosso, Francesca Malandrone, Sara Carletto, Chiara Benedetto

**Affiliations:** 1 Department of Clinical and Biological Sciences, University of Torino, Torino, Italy; 2 Clinical Psychology Service, City of Health and Science, Torino, Italy; 3 Gynecology and Obstetrics 1, Department of Surgical Sciences, City of Health and Science, University of Torino, Torino, Italy; 4 Department of Neuroscience “Rita Levi Montalcini”, University of Torino, Torino, Italy

**Keywords:** Depression, PTSD, Mental health, COVID-19, Pandemic, Pregnancy, Postpartum, Quarantine, SARS-Cov-2

## Abstract

**Background:**

Trauma, natural and man-made catastrophic events can be predictors of postpartum psychological distress. In a public health response due to coronavirus disease 2019 outbreak, the Italian government imposed a lockdown from March 9 to May 3. This extraordinary situation may have been challenging for maternal psychological health. The aim of this study was to investigate the prevalence of depressive and post-traumatic stress symptoms in women giving birth during the Covid-19 pandemic and its associations with quarantine measures, obstetrical factors, and relational attachment style.

**Methods:**

Women who gave birth in a high-volume obstetric/gynaecological medical centre located in an epidemic area during the Covid-19 pandemic (March 8 to June 15) were asked to complete an online survey about their childbirth experience and the perceived effect of the pandemic. The Edinburgh Postnatal Depression Scale (EPDS), the Impact of Event Scale-Revised (IES-R), and the Relationship Questionnaire (RQ) were administered to assess levels of postpartum depressive and post-traumatic stress symptoms (PTSS) and relational style of attachment, respectively. Multivariate analysis was applied to identify associations between quarantine measures, childbirth experience, attachment style, and EPDS and IES-R scores.

**Results:**

The survey was completed by 163 women (response rate 60.8%). The prevalence of depressive symptoms was 44.2% (EPDS cut-off score ≥ 11) and the PTSS rate was 42.9% (IES-R cut-off score ≥ 24). Dismissive and fearful avoidant attachment styles were significantly associated with the risk of depression and PTSS, respectively. Perceived pain during birth was a risk factor for postpartum depression. Perceived support provided by healthcare staff was a protective factor against depression and PTSS. Another protective factor against PTSS was quiet on the ward due to the absence of hospital visitors.

**Conclusion:**

This study reports a high prevalence of postpartum depressive and PTSS in women who gave birth during the Covid-19 pandemic. Postnatal psychological distress seemed to be associated more with the prenatal experience and other individual factors than with the pandemic hospital restrictions. Early detection during pregnancy of an insecure attachment style is fundamental to provide targeted preventive and therapeutic psychological interventions.

**Supplementary Information:**

The online version contains supplementary material available at 10.1186/s12884-020-03399-5.

## Background

Being female is the foremost risk factor for developing post-traumatic stress symptoms (PTSS) and depressive symptoms among adults and adolescents. Major stressors (e.g., health crises and natural disasters) can increase prenatal stress and make pregnant women particularly vulnerable [1, 2]. Mental health disorders are a common cause of morbidity during pregnancy, with approximately 12% of women experiencing depression and up to 22% experiencing high levels of anxiety in late pregnancy [3, 4]. Maternal distress during pregnancy has been associated with serious negative outcomes, including maternal psychosocial functioning, parenting difficulties, and offspring psychopathology [5, 6]. Childbirth can be experienced as a traumatic event owing to the presence of objective (e.g., obstetric complications) and subjective (e.g., loss of control, fear and pain during birth, lack of support) factors [7].

Previous studies reported that trauma, natural and man-made catastrophic events can be predictors of postpartum depression symptoms [8–14]. In February 2020, Italy became the epicentre of the coronavirus disease 2019 (Covid-19) outbreak in Europe. In a public health response, the Italian government imposed a lockdown (March 9 to May 3) and implemented restrictive measures such as social distancing, shutdown of activities, schools, and public places [15, 16].

Hospitals instituted visitor restriction policies that did not allow support persons, including the woman’s partner, to be physically present in obstetric maternity units, even during labour, except in the birth room. Pregnant women were no less affected than the general population; Covid-19 infection in epidemic areas was detected in about one out of ten women, regardless of the trimester of pregnancy [17, 18].

This extraordinary situation of isolation, loss of freedom, concern about the impact of Covid-19 on pregnancy or the possible vertical transmission of infection [19], and unfavourable obstetric outcomes may be challenging for maternal psychological health [20, 21]. Recent literature regarding the Covid-19 outbreak has largely focused on mental health and psychological needs during pregnancy. Following official statements on human-to-human transmission of severe acute respiratory syndrome coronavirus 2 (SARS-CoV-2), there was a clinically significant rise in the prevalence of depressive and anxiety symptoms among pregnant women in their third trimester [22–24].

The aim of the present study was to assess the prevalence and associated factors of postpartum depressive and PTSS during the Covid-19 pandemic. Our hypothesis was that specific factors related to the current pandemic may be associated with the rise in the incidence of depressive and PTSS.

## Methods

### Design and participants

For this cross-sectional study, the sample was composed of women who gave birth at the Obstetrics and Gynecology Unit 1, Sant’Anna Hospital, City of Health and Science, Torino, Italy during the hospital restrictions imposed by the Covid-19 pandemic. Study inclusion criteria were having given birth between March 8 and June 15 and age ≥ 18 years. The exclusion criterion was the inability to read/write Italian. The women were contacted either by telephone after discharge or in person while in hospital and asked for their email address by a resident in obstetrics and gynaecology. Those who agreed to participate in the study received an email with a link to a Google Form survey. All the questions in the survey were mandatory, in order to avoid missing data. Data were collected between June 15 and June 29. The study was conducted in accordance with the Declaration of Helsinki, and was approved by the Research Ethics Committee of the City of Health and Science, Torino, Italy. Informed consent was obtained by asking all participants to click a button at the beginning of the online survey to consent to participate.

### Study measures

The questionnaire contained items investigating sociodemographic factors (age, education, work status, living condition, nationality), obstetric factors (previous pregnancies, fertility treatments), their childbirth experience (level of pain experienced during childbirth, perceived level of support from health care staff during childbirth), and potential Covid-19 exposure, fear of contracting the virus, and discomfort/quiet experienced in the absence of their partner and other hospital visitors due to the restrictions in force (see Supplementary file 1 and 2).

To assess levels of postpartum depressive and PTSS and relational style of attachment, the following validated self-report questionnaires were administered:

- The Edinburgh Postnatal Depression Scale (EPDS) [25], a 10-item, four-point Likert-like scale questionnaire that assesses pregnancy and postpartum depression. The total score ranges from 0 to 30, with higher scores indicating more severe depression. A score between ≥11 and ≥ 13 is considered optimal for screening and detection of depressive symptoms, respectively [26–28].
- Impact of Event Scale-Revised (IES-R) [29], a 22-item questionnaire consisting of three subscales (8 items for intrusions, 8 for avoidance, and 6 for hyperarousal). The scale assesses subjective distress caused by traumatic events. For the present study, the women were asked to refer to their recent birth when responding. A score ≥ 33 is the best cut-off to identify moderate PTSS, while a score ≥ 24 indicates mild PTSS [30, 31].
- The Relationship Questionnaire (RQ) [32] is a single-item measure with four short paragraphs designed to measure adult attachment style. Each item describes a prototypical attachment pattern (secure RQ1, dismissive-avoidant RQ2, preoccupied RQ3, and fearful-avoidant RQ4) rated on a 7-point Likert-type scale. The dismissive avoidant, preoccupied, and fearful avoidant patterns are considered different forms of insecure attachment. The secure attachment pattern (RQ1) is described as: “It is easy for me to become emotionally close to others. I am comfortable depending on them and having them depend on me. I don’t worry about being alone or having others not accept me.” The dismissive-avoidant pattern (RQ2) is described as: “I am comfortable without close emotional relationships. It is very important to me to feel independent and self-sufficient and I prefer not to depend on others or have others depend on me.” The preoccupied attachment pattern (RQ3) is described as: “I want to be completely emotionally intimate with others, but I often find that others are reluctant to get as close as I would like. I am uncomfortable being without close relationships, but I sometimes worry that others don’t value me as much as I value them.” The fearful-avoidant attachment pattern (RQ4) is described as: “I am uncomfortable getting close to others. I want emotionally close relationships, but I find it difficult to trust others completely, or to depend on them. I worry that I will be hurt if I allow myself to become too close to others.” The highest score of the four attachment prototype ratings is used to classify individuals as having a predominant attachment style.

### Statistical analysis

Continuous variables are expressed as the mean ± standard deviation (SD) or median and interquartile range (IQR) when appropriate, while categorical variables are expressed as frequency and percentage. The Mann-Whitney or Student’s t test, Chi-square and Fisher’s exact tests were used to compare continuous and categorical variables. Multivariate analysis was carried out to identify associations with EPDS and IES-R. EPDS was dichotomized as 0 (absence of postpartum depressive symptoms) or 1 (presence of postpartum depressive symptoms) using a cut-off of 11 points. IES-R was categorized as 0 (absence of post-partum PTSS) or 1 (presence of PTSS) using a cut-off of 24 points. A multivariate model was developed based on statistical selection procedures. Variables were: pain level, support provided by healthcare staff during birth, attachment style, discomfort due to the absence of the partner, quiet on the ward due to hospital restrictions on visitors, days between and the date of birth and of questionnaire completion, discomfort due to Covid-19 before hospital admission, and if this was the first pregnancy. Model selection was performed using an automatic approach based on the Akaike information criteria (AIC) method. Given the large number of covariates, a genetic algorithm was employed to explore the candidate set of models. Model goodness of fit was evaluated with reference to the Brier score (the closer to 0, the better) and Somers’ Dxy Index, which indicates the ability of the model to discriminate. Odds ratios (ORs) and 95% confidence intervals (95% CIs) are reported. The significance level was set at *p* < 0.05. Statistical analysis was performed using R version 4.0.0.

## Results

Overall, 268 women were invited to participate in the online questionnaire; 163 of which completed the survey (60.8% response rate). Tables 1 and 2 present the sociodemographic and clinical characteristics of the sample, stratified by IES-R and EPDS. Regarding the RQ, it was not possible to determine the predominant style of attachment for five women. No differences were found between IES-R and EPDS categories, except for age. Women with postpartum post-traumatic and depressive symptoms were younger: the mean age of those with and those without distress was 33.6 and 35.7 years, respectively (*p* = 0.01); the mean age of those with and those without depression was 33.7 and 35.6 years, respectively (*p* = 0.015).

**Table 1:** Distribution of cohort demographics, overall and by presence of symptoms

	Overall	IES-R < 24	IES-R ≥ 24	***p-value***	EPDS < 11	EPDS ≥ 11	***p-value***
	***N*** = 163	***n*** = 93	***n*** = 70		***n*** = 91	***n*** = 72	
Age (years, mean ± SD)	34.77 (5.01)	35.65 (5.14)	33.61 (4.62)	0.010	35.62 (4.96)	33.71 (4.90)	0.015
Marital status (%)				0.100			0.164
Single	10 (6.1)	3 (3.2)	7 (10.0)		3 (3.3)	7 (9.7)	
Married/cohabitant	152 (93.3)	90 (96.8)	62 (88.6)		87 (95.6)	65 (90.3)	
Separated/divorced	1 (0.6)	0 (0.0)	1 (1.4)		1 (1.1)	0 (0.0)	
Level of education (%)				0.449			0.548
Low secondary school	16 (9.8)	8 (8.6)	8 (11.4)		7 (7.7)	9 (12.5)	
High secondary school	54 (33.1)	28 (30.1)	26 (37.1)		32 (35.2)	22 (30.6)	
University	93 (57.1)	57 (61.3)	36 (51.4)		52 (57.1)	41 (56.9)	
Employment status (%)				0.214			0.567
Unemployed	25 (15.3)	16 (17.2)	9 (12.9)		16 (17.6)	9 (12.5)	
Employed	135 (82.8)	77 (82.8)	58 (82.9)		74 (81.3)	61 (84.7)	
Student	1 (0.6)	0 (0.0)	1 (1.4)		0 (0.0)	1 (1.4)	
Partially employed	2 (1.2)	0 (0.0)	2 (2.9)		1 (1.1)	1 (1.4)	
Nationality (%)				0.135			0.162
Italian	148 (90.8)	86 (92.5)	62 (88.6)		82 (90.1)	66 (91.7)	
European	7 (4.3)	5 (5.4)	2 (2.9)		6 (6.6)	1 (1.4)	
non-European	8 (4.9)	2 (2.2)	6 (8.6)		3 (3.3)	5 (6.9)	

*IES-R* denotes Impact of Event Scale-Revised, *EPDS* Edinburgh Postnatal Depression Scale.

**Table 2:** Clinical data of the cohort, overall and by presence of symptoms

	Overall	IES-R < 24	IES-R ≥ 24	***p-value***	EPDS < 11	EPDS ≥ 11	***p-value***
	***N*** = 163	***n*** = 93	***n*** = 70		***n*** = 91	***n*** = 72	
First pregnancy (%)	74 (45.4)	37 (39.8)	37 (52.9)	0.134	36 (39.6)	38 (52.8)	0.127
Type of birth (%)				0.577			0.353
Vaginal	78 (47.9)	45 (48.4)	33 (47.1)		43 (47.3)	35 (48.6)	
Planned caesarean section	43 (26.4)	27 (29.0)	16 (22.9)		26 (28.6)	17 (23.6)	
Urgent caesarean section	32 (19.6)	17 (18.3)	15 (21.4)		19 (20.9)	13 (18.1)	
Forceps/vacuum	10 (6.1)	4 (4.3)	6 (8.6)		3 (3.3)	7 (9.7)	
Perceived support by healthcare staff during childbirth (median [IQR])	9 [7, 10]	10 [8, 10]	8 [6, 10]	0.002	10 [8, 10]	8 [6, 10]	0.002
Pain level during childbirth (median [IQR])	8 [2, 9]	7 [1, 9]	8 [5, 10]	0.156	7 [0.5, 9]	8 [5, 10]	0.036
Breastfeeding (%)	144 (88.3)	82 (88.2)	62 (88.6)	1.000	81 (89.0)	63 (87.5)	0.958
Confirmed diagnosis of Covid-19 (%)	5 (3.1)	3 (3.2)	2 (2.9)	1.000	1 (1.1)	4 (5.6)	0.237
Contact with Covid positive people (%)	8 (4.9)	7 (7.5)	1 (1.4)	0.156	5 (5.5)	3 (4.2)	0.980
Relatives/loved ones with a confirmed Covid-19 diagnosis (%)	21 (12.9)	13 (14.0)	8 (11.4)	0.807	11 (12.1)	10 (13.9)	0.916
Perceived safety during hospitalization (median [IQR])	8 [6;9]	8 [7, 9]	7.5 [6, 9]	0.385	8 [7, 9]	8 [6, 9]	0.340
Discomfort due to absence of partner	10 [8, 10]	10 [8, 10]	10 [9, 10]	0.009	10 [8, 10]	10 [8,75, 10]	0.315
Quiet on the ward related to the absence of visitors	7 [5, 8.5]	7 [6, 9]	6 [4, 8]	0.005	7 [5,9]	7 [5,8]	0.42
Time between childbirth and questionnaire completion ≤15 days (%)	25 (15.3)	15 (16.1)	10 (14.3)	0.917	15 (16.5)	10 (13.9)	0.812
Attachment style (%)				0.083			0.044
RQ1	65 (41.1)	42 (45.7)	23 (34.8)		44 (50.6)	21 (29.6)	
RQ2	60 (38.0)	37 (40.2)	23 (34.8)		30 (34.5)	30 (42.3)	
RQ3	8 (5.1)	4 (4.3)	4 (6.1)		3 (3.4)	5 (7.0)	
RQ4	25 (15.8)	9 (9.8)	16 (24.2)		10 (11.5)	15 (21.1)	

*IES-R* denotes Impact of Event Scale-Revised, *EPDS* Edinburgh Postnatal Depression Scale, *RQ* Relationship Questionnaire, *IQR* Interquartile range

Symptoms of postpartum depression were present in 72 (44.2%) women (EPDS cut-off ≥11) and in 50 (30.7%) (EPDS cut-off ≥13). Overall, symptoms of postpartum PTSS were present in 70 (42.9%) women (IES-R cut-off ≥24) and in 48 (29.4%) (IES-R cut-off ≥33). The RQ revealed that the majority reported an insecure attachment style: dismissive-avoidant in 60 (38%); fearful avoidant in 25 (15.8%); and preoccupied attachment pattern in 8 (5.1%) (Table 2).

Multivariate analysis of the EPDS (Table 3) showed a significant role for perceived pain: the risk of depression rose more than twice (OR 2.25, 95% CI 1.35–3.75; *p* = 0.002) for each 5-point increase on the scale assessing the level of pain experienced during childbirth. The relational attachment style was also found to be significantly associated with the risk of depression: women with an RQ2 attachment pattern had a significantly higher risk to develop depression than those with an RQ1 (OR 2.45, 95% CI 1.13–5.32; *p* = 0.024). Finally, the perceived support provided by healthcare staff during birth was a protective factor (OR 0.46, 95% CI 0.29–0.73; *p* = 0.01), indicating a risk reduction of depression of 54% for each 3-point increase on the perceived support scale. No significant association was observed between depressive symptoms and the quiet on the ward related to the absence of hospital visitors and the distress due to absence of the woman’s partner.

**Table 3:** Factors associated with postpartum depression and post-traumatic stress symptoms

*EPDS (cut-off score ≥ 11)*	**OR**	**95% CI**		***p-value***
Pain level during childbirth	2.254	1.354	3.754	0.002
Perceived support by healthcare staff during childbirth	0.460	0.289	0.730	0.001
RQ2 (vs. RQ1)	2.450	1.128	5.323	0.024
RQ3 (vs. RQ1)	3.680	0.728	18.607	0.115
RQ4 (vs. RQ1)	2.372	0.837	6.725	0.104
*IES-R (cut-off score ≥ 24)*	**OR**	**95% CI**		***p-value***
Distress related to the absence of partner	1.459	0.988	2.155	0.057
Quiet on the ward related to the absence of visitors	0.525	0.308	0.896	0.018
Perceived support by healthcare staff during childbirth	0.589	0.379	0.915	0.019
Time between childbirth and questionnaire completion	1.617	0.893	2.926	0.113
Pain level during childbirth	1.983	0.965	4.076	0.062
Fear of contracting Covid-19	1.484	0.989	2.225	0.056
First pregnancy	2.042	0.959	4.347	0.064
RQ2 (vs. RQ1)	1.273	0.556	2.917	0.568
RQ3 (vs. RQ1)	1.334	0.256	6.944	0.732
RQ4 (vs. RQ1)	3.651	1.188	11.219	0.024

*IES-R* denotes Impact of Event Scale-Revised, *EPDS* Edinburgh Postnatal Depression Scale, *RQ* Relationship Questionnaire, OR odds ratio, CI confidence interval.

The IES-R (Table 3) showed a significant association between the risk of developing postpartum PTSS and the attachment style. Women with an RQ4 attachment pattern had a higher risk than those with an RQ1 pattern. Finally, associated protective factors were the quiet on the ward because of the absence of visitors during hospitalization (OR 0.53, 95% CI 0.31–0.90; *p* = 0.018) and support by the healthcare staff during birth (OR 0.59, 95% CI 0.38–0.92; *p* = 0.019). The number of days between birth and questionnaire completion was included in the model as an adjusted covariate but had no significant association with depressive and post-traumatic symptoms.

## Discussion

The present study findings show that the prevalence of postpartum depressive and post-traumatic stress symptoms among the women experiencing childbirth during the Covid-19 pandemic was higher than that reported in previous studies before the pandemic. Literature data report that approximately 10–16% of women met major depression’s criteria at 3 months postpartum [24, 33–36]. The findings for our cohort (30.7%) are shared by a recent study that reported that 30% of the mothers who gave birth during the Covid-19 pandemic had a global EPDS score > 12 compared with 11.9% in an antecedent matched group of postpartum women [36]. An EPDS score > 13 was self-identified by another online survey in 15% of women before and in 40.7% during the outbreak for the same cohort of women who were pregnant or within the first year after birth [35].

Moreover, in our cohort 42.9% referred mild PTSS and 29.4% moderate symptoms. Previous studies investigating post-traumatic stress disorder (PTSD) rates after childbirth reported a prevalence rate of 3–4% in community samples and 15.7–18.9% in high-risk samples [37, 38]. Loss of control of oneself and excessive pain are the two most general elements of childbirth that make it potentially traumatising [39]. However, the psychological impact of the Covid-19 outbreak on pregnancy might explain the reported increase in PTSS also during the postpartum period.

The health status of the unborn child during the pandemic, the consequences of preventive measures, and the unmotivated fear of receiving less support and care during labour, birth or the pre and the post-natal period can all increase psycho-emotional distress. According to a recent survey, up to 95% of pregnant women reported mild PTSS and 61% moderate PTSS. More than two-thirds of the women also reported higher-than-normal anxiety, which was higher during the first trimester of pregnancy [23]. Data on the impact of coronaviruses on the first trimester of pregnancy are scarce; although no significant difference in the early abortion rate has been observed [40], viral infection at this stage could potentially affect embryogenesis and organ development.

In the present study, factors associated with postpartum depressive and post-traumatic symptoms were also investigated. Postpartum depressive symptoms were found to be associated with a high level of pain experienced during childbirth and an insecure dismissive-avoidant attachment pattern, while postpartum PTSS was associated with a fearful avoidant attachment style. The perceived level of support from the healthcare staff during childbirth was found to be a protective factor against the development of postpartum depressive and post-traumatic stress symptoms. Prior to the Covid-19 pandemic, the level of pain and perceived support were associated with postpartum depressive and post-traumatic symptoms [37, 41, 42]. An insecure attachment style was found to be significantly associated with depression and PTSD [43, 44], also in the perinatal period [45–50]. In our sample, a dismissive-avoidant attachment pattern was found to be significantly associated with postpartum depressive symptoms, while the fearful avoidant pattern was associated with PTSS. The dismissive-avoidant attachment pattern is characterized by a relational style that tends towards independence and autonomy. Individual and relational changes emerging during the perinatal period can conflict with the need for autonomy and the emotional difficulty to ask for relational (and psychological) support and the development of mother-infant bonding, which is an additional risk factor for postpartum depression [48]. The fearful avoidant attachment pattern is characterized by a combination of avoidant and anxious tendencies, low self-esteem, and the active search for intimate relationships and emotional closeness, without being able to trust other people.

The fearful avoidant profile seems to be more related to postpartum PTSS, as this attachment pattern is often present in people who have experienced previous relational trauma. Stress during the perinatal period might trigger a reactivation of traumatic memories, thus fostering the development of PTSD. Our findings are shared by a previous study that found fearful attachment to be associated with anxiety but not depressive symptoms in the immediate postpartum period [47]. Previous studies have also reported an association between the preoccupied attachment pattern and perinatal distress symptoms [50] which were absent in our study sample probably because of the few women in our cohort with this attachment style. An early evaluation of attachment style, which can be done during the prenatal period, could provide an additional strategy to identify women at are at higher risk to develop postpartum psychological distress and to offer them preventive interventions [45].

In our sample, the only Covid-19 related factor found to be significantly associated with symptoms during the postnatal period was the level of quiet on the ward due to the absence of visitors, which was a protective factor against the development of PTSS. The level of distress related to the absence of partners and the fear of contracting the virus approached statistical significance and so were not associated with depressive or post-traumatic symptoms. These findings may be interpreted in light of the higher prevalence of perinatal distress found in our and other studies conducted during the Covid-19 pandemic [22–24, 36]. The increase in postnatal distress seems to be related to symptoms present already during pregnancy. We may speculate that the increase in depressive and post-traumatic symptoms in women who gave birth during the Covid-19 outbreak may be related more to a general climate of alarm and concern about the pandemic than to specific factors with a direct impact on the childbirth experience. Future studies are needed to elucidate these associations and to evaluate the long-term impact of Covid-19 on the emotional distress of mothers and their relationship with children.

To our knowledge, this is the first study to evaluate depressive and PTSS and associated psychosocial factors during the postnatal period in women who experienced childbirth during the Covid-19 pandemic. The study has also some limitations. The lack of a pre-Covid-19 control group and of a psychological assessment during pregnancy may limit the generalization of postpartum prevalence data. Nevertheless, these factors do not seem to affect the primary aim of the study, which was to evaluate associated factors. Moreover, the potential bias of a retrospective survey was mitigated by including the variable “time since childbirth” as a covariate in the analysis.

## Conclusion

The study findings indicate a high prevalence of depressive and post-traumatic symptoms in the post-partum period in women who gave birth at a hospital located in an epicentre of the Covid-19 outbreak. Psychological distress was mainly associated with risk factors that are commonly reported in the literature, such as the level of pain experienced during birth, perceived support from healthcare staff, and attachment styles. The factors specifically related to the Covid-19 pandemic seemed to play an indirect role in increasing psychological distress. A future area of focus is to investigate their role.

Early detection of distress, which includes evaluation of psychological factors such as the attachment style, is fundamental for specific and targeted psychological interventions to contrast the negative impact that postpartum depression and PTSD can have on women’s psychosocial health, mother-infant bonding, and child development.

## Supplementary Information

**Additional file 1:** **Supplementary file 1.** Survey developed for the study – English language version.

**Additional file 2:** **Supplementary file 2.** Survey developed for the study – Original language version (Italian).

## Data Availability

The datasets generated during and/or analysed during the current study are openly available in the GitHub repository (https://github.com/berkeley3/covid19_SantAnna).

## Abbreviations

PTSS: Post-traumatic stress symptoms
Covid-19: Coronavirus disease 2019
SARS-COV-2: Severe acute respiratory syndrome coronavirus 2
EPDS: Edinburgh Postnatal Depression Scale
IES-R: Impact of Event Scale-Revised
RQ: Relationship Questionnaire
SD: Standard deviation
IQR: Interquartile range
AIC: Akaike information criteria
ORs: Odds ratios
95% CIs: 95% confidence intervals
PTSD: Post-traumatic stress disorder
