# Supplementary material for: Psychosocial factors associated with postpartum psychological distress during the Covid-19 pandemic: a cross-sectional study
Source: BMC Pregnancy Childbirth. 2020 Nov 18;20:703. doi: 10.1186/s12884-020-03399-5 (PMC7671935; doi:10.1186/s12884-020-03399-5)
Supplement: Supplementary file 1 — Additional file 1: Supplementary file 1. Survey developed for the study – English language version. [file 12884_2020_3399_MOESM1_ESM.docx]

**Supplementary file 1 – Survey developed for the study – English language version**

*Dear Patient,*

*You are invited to participate in our study on the impact the Covid-19 emergency has had on your experience of pregnancy, delivery, and stay in the Maternity Ward of the Sant’Anna Hospital, A.O.U. Città della Salute e della Scienza. This brief online questionnaire asks general questions and more specific ones about your pregnancy, delivery of your baby, hospital stay, and psychological well-being. It takes 15-20 minutes to complete. Your participation in the study is voluntary, anonymous, and free of charge. There are no foreseeable risks associated with participation. You can stop at any time if you do not wish to respond to a question. You can withdraw your consent to participate at any time without consequences. Should you have questions about the survey, you can contact the study researchers in writing at the following email address: Dr. XXXXX. The questionnaire is anonymous and conforms with ethical research standards. The study is conducted according to the tenets of the Declaration of Helsinki. Personal data are protected under the provisions of the data protection law (Decree Law 101/18) and by the study coordinators. Personal data required for the study are collected, treated (anonymously), and stored according to current norms (Decree Law no. 196 dated 30 June 2003, Guidelines for the Treatment of Personal Data in Clinical Studies, no. 52 dated 24 July 2008, General Data Protection Regulations [GDPR] 20/679, Decree Law 101/2018). The data collected for this study will be accessible to the qualified staff of the health care agency, the promotor, or its delegates and ethical committee and will respect the rights of the study subjects without breach of confidentiality of data within the limits of current legislation.*

*Thank you for participating in the study.*

- I have read and understood the above conditions and grant consent to participate in the study.
  - Yes/No
- Informed consent: You can start the survey by ticking the “Consent” box and then clicking on the “Next” key at the bottom.
  - I consent/I do not consent

Attach Personal Data Treatment Authorization

**DATA COLLECTION FORM**

Date……………..

**Demographics**

- Age:
- Marital Status
  - Single
  - Married/Cohabitant
  - Separated/Divorced
- Highest level of education:
  - Primary school
  - Low secondary school
  - High secondary school
  - University
- Employment status
  - Unemployed
  - Employed
  - Partially employed
  - Student
- Nationality
  - Italian
  - European
  - non-European
- How many pregnancies did you have before the current one?
  - None, it’s the first pregnancy
  - 1
  - 2
  - 3
  - 4
  - 5
  - More than 5

**Questions about the delivery of your child**

- Delivery date: ……………………………
- Type of delivery: what kind of delivery did you have?
  - Vaginal
  - Planned caesarian section
  - Urgent caesarian section
  - Forceps/vacuum
- Pain level experienced during labor and childbirth:

0…….….1............ 2.............3............ 4............... 5............. 6.............7…….....8……..…9………..10

None   Worst imaginable

- Perceived support from the midwife during labor and childbirth (or other health care staff):

1............. 2.............3............. 4.............. 5............... 6..............7………....8……....…9……………..10

None High

**Breastfeeding**

- Type of breastfeeding:
  - Only breast
  - Breast and bottle (mixed)
  - Only bottle

**The following questions concern your experience during the COVID-19 emergency.**

- Confirmed diagnosis of Covid-19
  - Yes
  - No
  - I prefer not to answer
- Contact with Covid positive people
  - Yes
  - No
- Relatives/loved ones with a confirmed Covid-19 diagnosis
  - Yes
  - No

**During hospital stay**

- Discomfort due to absence of partner

1............. 2.............3............. 4.............. 5............... 6..............7………....8……....…9……………..10

Not at all   Very stressful

- Perceived safety during hospitalization

1............. 2.............3............. 4.............. 5............... 6..............7………....8……....…9……………..10

Not at all   Very protected

- Quiet on the ward related to the absence of visitors

1............. 2.............3............. 4.............. 5............... 6..............7………....8……....…9……………..10

Not at all   Very much
