# Supplementary material for: Psychosocial factors associated with postpartum psychological distress during the Covid-19 pandemic: a cross-sectional study
Source: BMC Pregnancy Childbirth. 2020 Nov 18;20:703. doi: 10.1186/s12884-020-03399-5 (PMC7671935; doi:10.1186/s12884-020-03399-5)
Supplement: Supplementary file 2 — Additional file 2: Supplementary file 2. Survey developed for the study – Original language version (Italian). [file 12884_2020_3399_MOESM2_ESM.docx]

**Supplementary file 2 – Survey developed for the study – Original language version (Italian).**

*Gentile Signora,*

*La invitiamo a partecipare al nostro studio finalizzato a rilevare l'impatto che l’emergenza sanitaria COVID-19 ha avuto sulla Sua esperienza di gravidanza, parto e ricovero presso il reparto maternità del P.O. Sant’Anna, A.O.U. Città della Salute e della Scienza.*

*Le chiediamo di compilare una breve indagine online, che include sia domande generiche, sia domande relative alla Sua gravidanza, parto e periodo di ricovero in Ospedale e al Suo stato psicologico. Questa indagine richiederà all’incirca 15/20 minuti per la sua compilazione.*

*La Sua partecipazione è completamente volontaria, in forma anonima e gratuita. Non ci sono rischi prevedibili associati a questo studio. Tuttavia, se si sentisse a disagio nel rispondere ad una qualsiasi domanda, può interrompere l'indagine in qualunque momento. Potrà inoltre ritirare il consenso in qualsiasi momento, senza alcuna conseguenza. Se dovesse avere delle domande riguardo l'indagine, può contattare i ricercatori coinvolti ai seguenti indirizzi e-mail: Dott.* *XXXXX.*

*Il questionario è anonimo e rispetta tutti gli standard etici della ricerca. Lo studio è condotto in accordo con i principi etici sanciti dalla Dichiarazione di Helsinki. In particolare, la protezione dei dati personali è tutelata sia dalla specifica normativa (D.L. 101/18) sia dalle persone responsabili del progetto di ricerca. I dati personali necessari per lo studio verranno raccolti e trattati (in maniera completamente anonima) e conservati nel rispetto della normativa relativa (D.Lgs. 30 giugno 2003 n. 196, Linee Guida per il trattamento di dati personali nell’ambito delle sperimentazioni cliniche del. N.52 del 24 luglio 2008, GDPR 2016/679, D. Lgs. 101/2018). I dati relativi allo studio saranno resi disponibili al personale qualificato della Autorità Sanitaria, del Promotore o di suoi Delegati e dei Comitati Etici nel totale rispetto dei diritti dei soggetti coinvolti e senza violare la confidenzialità dei dati nella misura consentita dai regolamenti di legge.*

*La ringraziamo anticipatamente per il Suo tempo e per la Sua collaborazione.*

- Ho letto, compreso e acconsento alla partecipazione allo studio
  - Si/No
- Consenso informato: può iniziare l'indagine selezionando la casella “Acconsento” e cliccando successivamente sul pulsante “Avanti” in basso.
  - Acconsento/Non acconsento

**MODULO RACCOLTA INFORMAZIONI**

Data……………..

**Anagrafica**

- Età:
- Stato Civile
  - Single
  - Sposata/Convivente
  - Separata/Divorziata
- Titolo di studio
  - Scuola elementare
  - Licenza media inferiore
  - Licenza media superiore
  - Laurea
- Professione attuale
  - Disoccupata
  - Impiegata
  - Occupazione part-time
  - Studentessa
- Nazionalità
  - Italiana
  - Europea
  - extra Europea
- Ha mai avuto altre gravidanze prima di quella in corso?
  - Nessuna, è la prima
  - 1
  - 2
  - 3
  - 4
  - 5
  - Più di 5

**Domande relative al parto**

- Data del parto: ……………………………
- Tipo di parto: che tipo di parto ha avuto?
  - Vaginale
  - Parto cesareo programmato
  - Parto cesareo urgente
  - Parto con forcipe/ventosa
- Livello di dolore esperito durante il parto:

0…….….1............ 2.............3............ 4............... 5............. 6.............7…….....8……..…9………..10

Nessuno   Moltissimo

- Livello di supporto percepito durante il parto da parte dell’ostetrica (ed eventuale altro personale sanitario):

1............. 2.............3............. 4.............. 5............... 6..............7………....8……....…9……………..10

Per nulla Moltissimo

**Allattamento**

- Tipo di allattamento:
  - Al seno esclusivo
  - Al seno con aggiunta di latte artificiale (misto)
  - Artificiale completo

**Di seguito le rivolgiamo alcune domande inerenti alla sua esperienza durante l'emergenza sanitaria Covid-19.**

- Diagnosi confermata di Covid-19
  - Si
  - No
  - Preferisco non rispondere
- Contatti con persone positive al Covid-19
  - Si
  - No
- Persone a lei vicine con diagnosi confermata di Covid-19
  - Si
  - No

**Durante il ricovero**

- Disagio dovuto all’assenza del partner

1............. 2.............3............. 4.............. 5............... 6..............7………....8……....…9……………..10

Per niente   Moltissimo

- Quanto si è sentita al sicuro in reparto

1............. 2.............3............. 4.............. 5............... 6..............7………....8……....…9……………..10

Per niente   Moltissimo

- Apprezzamento della tranquillità legata alle restrizioni nelle visite dei suoi visitatori

1............. 2.............3............. 4.............. 5............... 6..............7………....8……....…9……………..10

Per niente   Moltissimo
